# *In vitro* fertilisation in domestic mammals—a brief overview

**DOI:** 10.1080/03009734.2019.1697911

**Published:** 2019-12-13

**Authors:** Ylva Sjunnesson

**Affiliations:** Department of Clinical Sciences, Reproduction, The Centre for Reproductive Biology in Uppsala (CRU), Swedish University of Agricultural Sciences (SLU), Uppsala, Sweden

**Keywords:** Embryo, in vitro maturation, in vitro production, oocyte, species comparison

## Abstract

Many factors influence the final oocyte maturation, fertilisation, and early embryo development, and there are both similarities and differences between species. When comparing the advancement of assisted reproductive technologies (ARTs), the development in the bovine species is not far behind the medical front, with around one million *in vitro*-produced bovine embryos each year. This rate of progress is not seen in the other domestic species. This review aims to give an overview of the development and specific difficulties of *in vitro* embryo production in various domestic animal species, with the main focus on cows, pigs, and cats. In production animals, the aim of ARTs is commonly to increase the genetic progress, not to treat reproductive failure. The ARTs are also used for preservation of genetic diversity for the future. However, specifically for oocyte maturation, fertilisation, and early embryonic development, domestic mammals such as the cow and pig can be used as models for humans. This is particularly attractive from an animal welfare point of view since bovine and porcine oocytes are available in large numbers from discarded slaughterhouse material, thereby decreasing the need for research animals. Both for researchers on the animal and human medical fronts, we aim for the development of *in vitro* production systems that will produce embryos and offspring that are no different from those conceived and developed *in vivo*. Species-comparative research and development can provide us with crucial knowledge to achieve this aim and hopefully help us avoid unnecessary problems in the future.

## Introduction

It is easy to assume that in the beginning of our embryonic development we animals are all very much alike. In some aspects this is true, but there are major differences between species, and even between mammals. In many ways, cows and humans are similar in the advancement of assisted reproductive technologies (ARTs) and *in vitro* fertilisation (IVF), but many species still lag far behind. This review attempts to give an overview of the development and specific difficulties of *in vitro* embryo production, including IVF, in various domestic animal species, with the main focus on cows (bovines), pigs (porcines), and cats (felines). Commonly in production animals, the focus is not to overcome reproductive failure but to enhance the genetic progress, both by selection of the most genetically valuable individuals and by increased number of offspring from these individuals. With species-comparative knowledge, our possibilities to understand the mechanisms behind various aspects of the final oocyte maturation, fertilisation, and early embryonic development *in vitro* will increase. IVF-associated ARTs such as artificial insemination, which is used successfully and to a very large extent in developed countries, sperm capacitation, intracytoplasmic sperm injection (ICSI), embryo transfer (ET), and cloning fall outside the scope of this review and are only briefly mentioned.

## An overview of procedures

**Figure 1.**
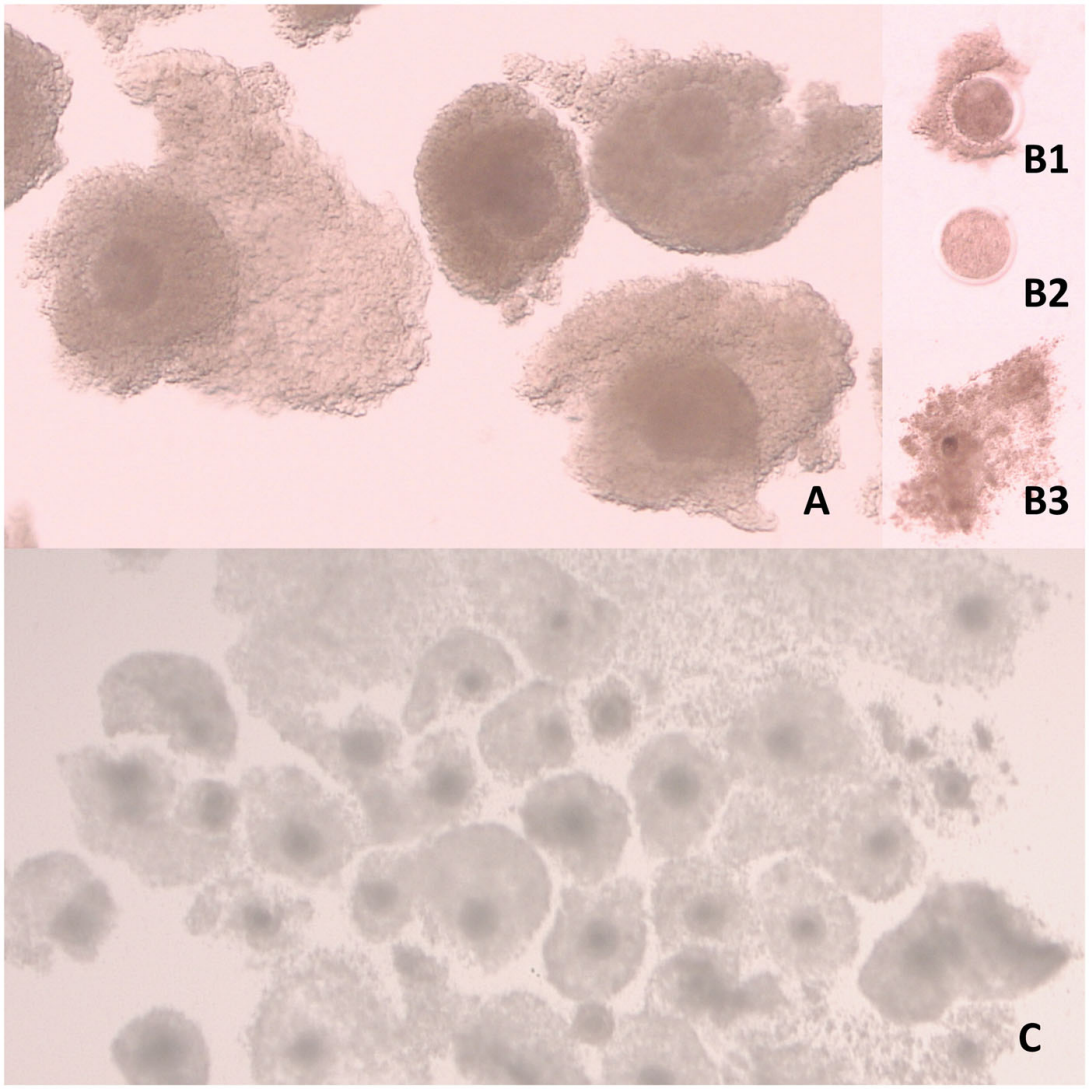
Bovine oocytes. (A) Immature oocytes of acceptable quality for IVP. (B) Immature oocytes of unacceptable quality for IVP (B1: partially nude oocyte; B2: completely nude oocyte with pale cytoplasm; B3: over-expanded oocyte). (C) Oocytes matured *in vitro* where the majority have good expansion of cumulus cells.

**Figure 2.**
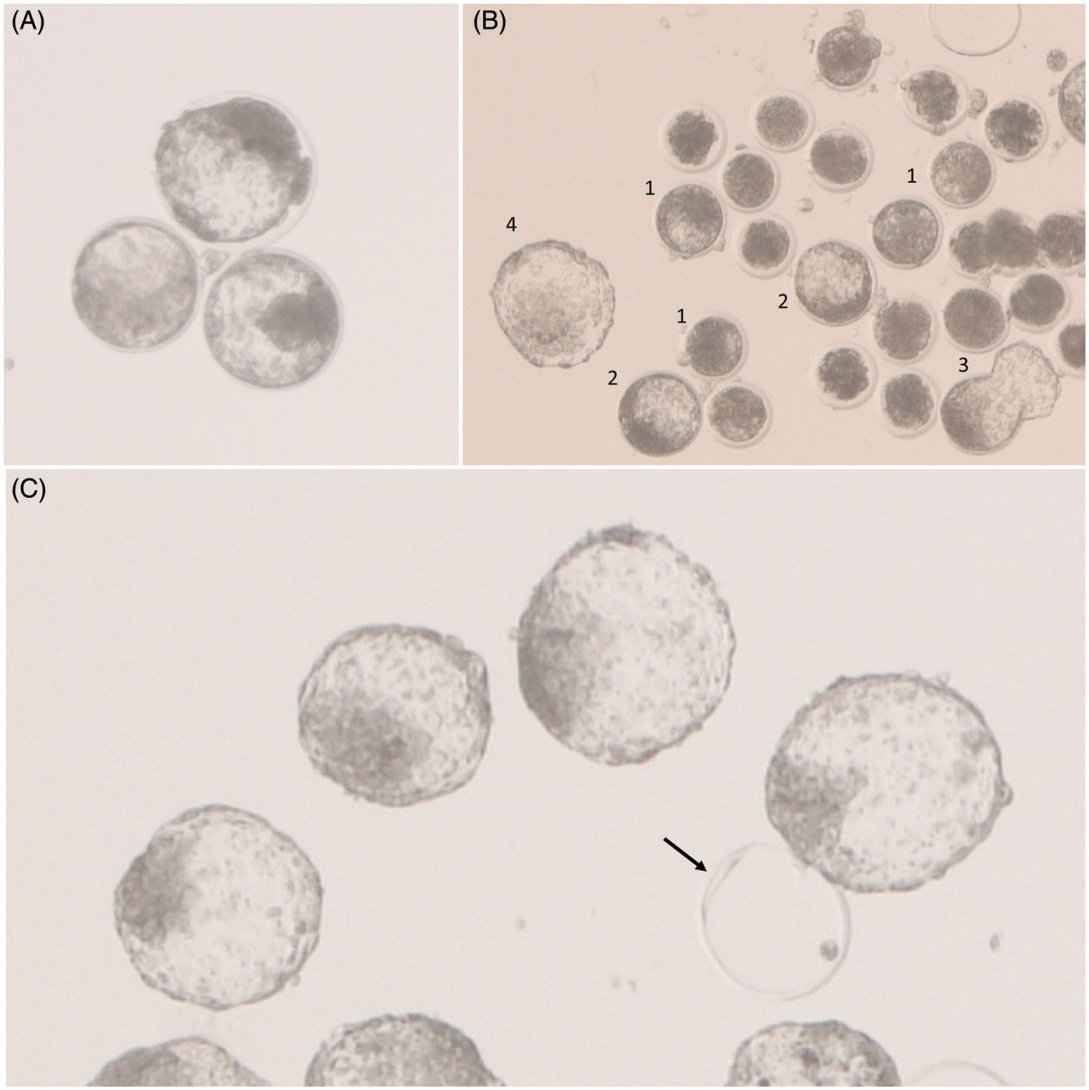
*In vitro*-produced bovine embryos day 8 after fertilisation. (A) Expanded blastocysts. (B) Mix of blastocysts, embryos in earlier developmental stages, and non-fertilised oocytes (1: blastocysts; 2: expanded blastocysts; 3: hatching blastocyst, 4: hatched blastocyst). (C) Hatched blastocysts of good quality. The arrow indicates an empty zona pellucida after hatching.

The procedures of *in vitro* oocyte maturation (IVM), *in vitro* fertilisation (IVF), and the first days of *in vitro* embryo culture (IVC) are collectively termed *in vitro* embryo production (IVP). In common domestic animals we usually retrieve immature oocytes from the ovaries, either after normal castration where ovaries are removed, in ovum pick-up (OPU) procedures in live animals, or after the death of the animal (due to slaughter, euthanasia, or after fatal trauma). In experimental settings or within some commercially IVP companies, *in vivo* maturation procedures may occur. Since animal ovaries are not considered fit for human consumption in most countries, they are a waste product in the meat industry and can easily be retrieved fresh in large quantities from consenting abattoirs. Routine castrations or neutering/spaying of pets are performed on the explicit wishes of the owners and according to recommendations of veterinarians due to various reasons that can include prevention of unwanted offspring as well as changes in temperament or behaviour connected to hormonal actions in an intact animal (1). Normal routine castrations are performed daily in most veterinary clinics, and also here the ovaries are discarded as waste and can be collected for IVP purposes (2). This relatively easy access to large quantities of ovaries without having to use research animals makes species like the cow and pig perfect candidates for IVP research, spanning from testing of *in vitro* media and methodological improvements to controlled trials of newly emerging risk substances like environmental pollutants found in humans and our environment (3,4). However, with the use of these discarded ovaries there are no possibilities to control cyclicity or follicular growth that would be possible in research animals. Due to the presence of continuous follicular waves in most domestic animals (5), the ovaries will always contain cumulus oocyte complexes (COCs). Therefore, the approach is usually to retrieve relatively immature COCs and perform the final maturation *in vitro*. The selection process starts with aspiration of follicles of a certain size range depending on species, commonly by hand with needles and syringes. The follicles aspirated should not be too small, since they would contain COCs that are too immature and difficult to mature to MII stage (6,7). On the other hand, the follicles should not be too large since they could contain COCs that are too mature, with already-expanded cumulus cells or atretic oocytes, that would make them unsuitable to include in the process (Figure 1). After the aspiration or OPU all COCs are collected, and only COCs of good quality are selected for the IVM process (Figure 1). The ideal bovine COC is light and transparent, and has a compact multilayered cumulus investment and a homogeneous ooplasm (8). In both pigs and cats the COCs are darker than in the bovine, but otherwise the same criteria are used. During IVM, media are supplemented with LH and FSH, and in some cases oestradiol, growth hormones, and insulin. After IVM the medium is changed to promote sperm capacitation and fertilisation. The duration of fertilisation ranges between a few hours and up to one day, depending on species, and is followed by IVC where the medium is promoting the early embryo development. Embryos are commonly evaluated and classified according to the International Embryo Technology Society (IETS) guidelines (9). A common time point for transfer back to a recipient animal is in the stages of morula or early blastocyst, but later blastocyst stages as well as earlier embryos could also be transferred (Figure 2). In order to avoid the risk of transferring diseases, embryo transfer teams (mainly working with bovines) must also follow the recommendations of the IETS (10).

## History of IVP in domestic mammals

Already in the 1950s, live births after IVF were demonstrated in rabbits and, one decade later, in mice (Table 1). In the beginning, successful IVP systems were usually *in vivo*-based co-culture systems where maturation/fertilisation and/or culture took place in oviducts from rabbit or sheep and later on after co-culture with oviductal cells. Gradually, supporting cells were excluded as formulas for the media improved. Chemically defined media have given valuable insights to our understanding of the impact of various substances on oocyte maturation, fertilisation, and early embryo development and the possibility to avoid unknown macromolecules. Moreover, potential contaminants from e.g. serum in culture conditions are of great importance (33). ARTs have over the years developed from invasive surgical procedures to ultrasound-guided techniques in many species. IVM is still a major hurdle to overcome in most species, and another difficulty has been the capacitation process. Different species, as well as male individuals within the same species (34), have differences in substances and concentrations of these that can induce capacitation. Heparin, serum albumin, epinephrine, penicillamine, hypotaurine, coffein, bicarbonate, and calcium are some of the substances that can induce capacitation (35,36). It is quite obvious that there is a large step from maturing and fertilising oocytes *in vitro* to actually being able to produce live offspring after both procedures since this seems to take over 20 years in most species (Table 1). It is worthy of note that, even though cases of successful IVF with live offspring have been reported in dog and horse, such reports have been few and have proven difficult to repeat.

**Table 1.** Examples of years of early attempts of IVM, IVF, and IVC in selected species.

Species	IVM (ref.)	IVF (ref.)	Birth of live offspring after IVF (ref.)	Birth of live offspring after IVM and IVF (and in some cases IVC) (ref.)
Cattle	1965 (11)	1977 (12)	1982 (13)	1990 (14)
Pig	1965 (11)	1975 (15)	1986 (16)	1989 (17)
Sheep	1965 (11)	1959 (18)	1986 (16)	1986 (16)
Rabbit	1955 (19)	1954 (20)	1959 (21)	1983 (22)
Mouse	1965 (11)	1968 (23)	1970 (24)	1970 (24)
Cat	1989 (25)	1989 (25)	1988 (26)	1997 (27)
Dog	1976^a^ (28)	1976^a^ (28)	2015^a^ (29)	
Horse	1981^a^ (30)	1989^a^ (31)	1991^a^ (32)	

^a^Limited success.

## IVP in cattle

Today the cattle IVP industry is expanding at a high rate and does not show any signs of slowing down. According to the International Embryo Technology Society (IETS) almost one million transferable *in vitro-*produced cattle embryos were produced all over the world in 2017 (Table 2). Unfortunately, not all countries contribute to this report, and these numbers are therefore under-estimated. There has been a remarkable increase since 2016 when only about half a million bovine embryos were produced. Most of the transfers were carried out in Brazil, closely followed by the USA. In Europe, there were some 50,000 transfers of IVP embryos carried out, and most of them were done in Russia followed by the Netherlands, Spain, Germany, and France. The total number of embryo transfers (both of IVP and *in vivo*-derived embryos) all over the world was over 1.5 million in 2017 (37).

**Table 2.** Summary of IVF in common domestic mammals.

Species	Main purpose of IVF	Approximated number of *in vitro*-produced embryos in 2017	Examples of specific characteristics
Bovines	Genetic progress	1,000,000^a^	Most techniques used in humans work well except ICSI
Porcines	Research and genetic modifications	Limited number and not possible to estimate	Prone to polyspermia *in vitro*, and gametes are highly sensitive to low temperatures
Felines	Model for endangered feline species	Very limited number and not possible to estimate	Induced ovulators
Equine	Increasing reproductive potential among selected individuals	500^a^	IVF is not successful, but ICSI and cloning are performed
Canine	Research	Extremely limited number and not possible to estimate	Oocytes are in MI stage at ovulation and difficult to mature *in vitro*
Ovine	Genetic progress	100^a^	Surgical ovum pick-up and transfer are needed

^a^Reference (37).

Bovine IVP systems are working very well, but there is always room for improvement. Approximately 90% of the aspirated oocytes reach MII stage, and some 80% of these become fertilised *in vitro*. After culture *in vitro* around 50% of the fertilised oocytes become blastocysts (38).

The success story of the cattle IVP could have been very different due to the appearance of the large offspring syndrome (LOS) in the late 1980s and the following decade. After transfer of IVP embryos, alarming reports started to emerge, beginning in the farming press, where some of the calves born had malformations and were too large, which led to difficult calvings (33,39). Naturally, this became a serious problem for animal welfare, and researchers searched for the background mechanism and a solution. Even at present, the full mechanisms are unknown, but the addition of serum to the culture medium has been identified as a likely factor behind the LOS in both sheep and cows (40). Today bovine serum albumin and amino acids are used during culture, instead of serum, with better results. It seems likely that the effects of the serum were through epigenetic changes (41). One of the lessons to be learned from the LOS legacy is that seemingly minor changes in medium composition may have a pronounced impact on the offspring.

OPU has been modified for successful use in cattle. The technique in cattle is supported by the possibility of the operator to move an ovary to the right position via a gloved hand in the rectum of the cow. It is the major technique to retrieve oocytes for IVP and ET (37). In the Swedish University of Agricultural Sciences (SLU), Uppsala, extensive studies have been carried out to investigate the animal welfare effects of repeated OPU sessions on heifers (42), the only negative experience seemingly being the routine administration of the epidural anaesthesia needed.

Even though many techniques used in humans have worked very well in cattle, the ICSI procedure is still far from successful, possibly because no spontaneous activation of the oocyte occurs after injection of a spermatozoon (43). However, it could also be attributed to other factors such as the darkness of the ooplasm, the large sperm heads, and the toughness of the oolemma. This is in contrast to other forms of micromanipulation that seem to work nicely in cattle. Micromanipulation of bovine embryos for sexing and/or genotyping is becoming more and more common, with around 10,000 embryos tested in 2017 (37). The number is likely to have increased since 2017 when more relevant genetic information for selection of the best possible traits in cattle rapidly became available. This possibility to make fast genetic progress also increases the use of IVP in cattle.

## IVP in pigs

The production of pig embryos *in vitro* still has many problems to overcome before it can be said to be efficient. One of the main problems is inherent to IVF itself, which often results in polyspermic fertilisation. When the sperm concentration is decreased, there is just a minor effect until the concentrations are so low that there will be no penetration of spermatozoa at all. A failure of the pig oocyte maturation *in vitro* is probably connected to the polyspermic situation (44), but other factors such as an altered zona reaction (45), and even temperature during IVF (46), have been proposed. The pig IVP has a two-day maturation process, and it is likely that there are many conditions having an impact on oocyte maturation that we are not aware of as yet. IVM in pigs gives relatively high rates of oocytes matured to MII stage, between 75% and 85%, but unfortunately the polyspermic rates can reach 50–70% (47). This was observed in the first experiment using single-layer colloid centrifugation before pig IVF was performed in the IVP unit at SLU, Uppsala (48). To overcome the extreme fertilising efficiency of the colloid centrifugation-selected spermatozoa, the concentrations of spermatozoa for fertilisation had to be lowered from the standard 5 × 10^5^ spermatozoa/mL to 4 × 10^3^ spermatozoa/mL. Since polyspermic porcine embryos can cleave and develop to the blastocyst stage, as normal embryos (49), polyspermia has to be assessed by investigation of pronuclei. This has to be done via staining or high-*g* centrifugation in most domestic animals since the ooplasm is too dark to visualise the pronuclei in light microscopy. If the oocytes are monospermic, around 80% can reach the blastocyst stage (47). Recent research within this field is mostly focussed on selecting spermatozoa in various ways to avoid polyspermy (50). ICSI is used with some success in pigs (51), thereby bypassing the polyspermy problem entirely.

Apart from the polyspermy problem, pig oocytes and embryos are very sensitive to low temperatures. If temperature drops below 25 °C the oocyte maturation process permanently stops in most cases (52). With pig embryos, it is not possible to store them at a low temperature before transfer since they are all irreversibly damaged if temperature drops below 10 °C (53). Furthermore, porcine oocytes and embryos, compared with other mammalian species, are very sensitive to cryopreservation, possibly due to the high amount of lipids present (54).

The advantages of an efficient pig IVP are many. The genetical and technical progress could of course be significantly secured and enhanced, but the pig also has a high value as a model for human situations. Despite many difficulties, IVP in pigs is continuously explored. Research in Uppsala led to the first production of piglets after fertilisation and culture in chemically defined media where the effects of various components during fertilisation were assessed (55).

## IVP in cats

No doubt, the domestic cat population as a whole has no problem to reproduce. However, the domestic cat is an excellent model for other feline species, and almost all large cats are threatened by extinction at least to some extent. Only a few domestic kittens have been born after IVF (26,27,56). When a genetically important animal unexpectedly dies, gametes can be collected and either directly subjected to IVP or frozen for future use. For cats, between 40% and 80% of the COCs mature to MII phase, and the proportion of cleaved embryos developing *in vitro* can reach 70% (56) but is usually lower. In SLU Uppsala, we have used the domestic cat as a model to increase the number of spermatozoa that can be salvaged from the testicles after removal. The standard procedure is to collect only spermatozoa from the cauda epididymis since this is where the most mature spermatozoa can be found (57). Using an IVP system, we found that spermatozoa from the corpus epididymis had fertilising capacity and blastocysts were produced, even if it was at a lower rate (2). With this feline model we saw around 8% parthogenetically activated oocytes, but none continued to the blastocyst stage (unpublished data). Since the parthogenic embryos can be similar to normal embryos, they cannot be separated by simple microscopy.

## IVP in horses, dogs, and sheep

Even though this short review cannot cover all domestic mammals where IVF has been performed or attempted, some common species have to be mentioned.

IVF methodology in the horse has so far never been well established, but ICSI and cloning have been done with good results (58). The reason for the failed IVF in the horse has been extensively discussed by Leemans et al. (59) and is believed to be due to the failure to capacitate stallion spermatozoa. The interest for IVF and related technologies for the horse is increasing within certain sports and breeds where money is available and reproductive biotechnologies are allowed. Unfortunately, mares seem to be poor responders to superovulation (60), and only one or few oocytes can commonly be retrieved per cycle; this, together with the lower access to slaughterhouse ovaries, makes progress in this field slow. The total number of *in vitro*-derived embryos transferred in the horse is currently low (Table 2). However, the numbers can be expected to rise in the coming years as ICSI, and possibly also cloning, become more established.

Dogs pose a special case since their oocytes are ovulated in the MI stage and several days are required for them to mature to the MII stage in the oviduct. *In vitro* maturation has never been truly established, but after *in vivo* maturation and *in vivo* culture the first—and, so far, only—puppies were born after IVF in 2015 (29). The main hurdles for dog IVP are the maturation process and the fact that dogs are mono-oestroual breeders, which means that the oestrus is followed by a long pause, resulting in a bitch normally only having one to three oestrous periods during one year.

*In vitro* production of sheep embryos is well established, but success rates are lower than for bovines (61). Many factors regarding sheep IVP correlate well to cows as they are both ruminants. Unfortunately, surgical OPU and transfer are still necessary in sheep due to their anatomy, and this makes the procedure far more invasive than in bovines. The commercial activity of ovine *in vitro*-derived embryo transfer is very low (Table 2).

## Animals as models for humans

In many cases animals can be used as models for humans with regard to the final oocyte maturation, fertilisation, and early embryo development *in vitro*. Depending on which factors are to be investigated, different species might be of interest. Thus, rodents are not always the most suitable models for humans, especially when considering oocyte maturation and fertilisation (62). One example is the oocyte transcriptome where human oocytes are more closely related to cow oocytes than to mouse oocytes (63). There are also many other specific characteristics making the human, the cow, and the pig much more similar compared with rodents, for example the timing of the early embryo development *in vitro* (4).

In the IVP unit for pigs in SLU Uppsala, the impact of stress was investigated by adding plasma from stressed or non-stressed sows to the IVP media (64,65). Plasma was collected from the corresponding time points around ovulation *in vivo*, and stress was simulated by adrenocorticotropic hormone injections. In these studies, stress seemed to have a major impact on spermatozoa function at the time of fertilisation, possibly by prematurely induced acrosome reactions (65).

In the SLU Uppsala IVP unit for cows, several studies have been conducted with focus on the impact of insulin during IVM (66). These studies were mainly focussed on the metabolic imbalance seen in dairy cattle after calving but are also of comparative interest to metabolic disorders in humans. We observed a negative impact of insulin in IVM on blastocyst development, evidenced by an effect on the gene expression patterns as well as morphologically.

As a final example, LOS should be mentioned. Since LOS can be induced by the addition of serum to culture medium, it has been proposed as a model for Beckwith–Wiedemann syndrome in human (67). So, hopefully this major setback can be made useful in future research.

## Future development

With the arrival of new and technically relatively simple tools for gene edition—CRISPR-Cas9 and TALEN systems (for reviews, see 68,69)—there is an increased interest in genetic modifications of animals aimed for both production and research.

Given the new possibilities for genetic modification and the physiological similarities between humans and pigs, the pig is regarded as an excellent model for human diseases, studies of gene functions, as well as pharmacological studies (68,69). Potentially, the genetically modified pig could also be a donor of tissues and organs for xenotransplantation (47).

**Figure 3.**
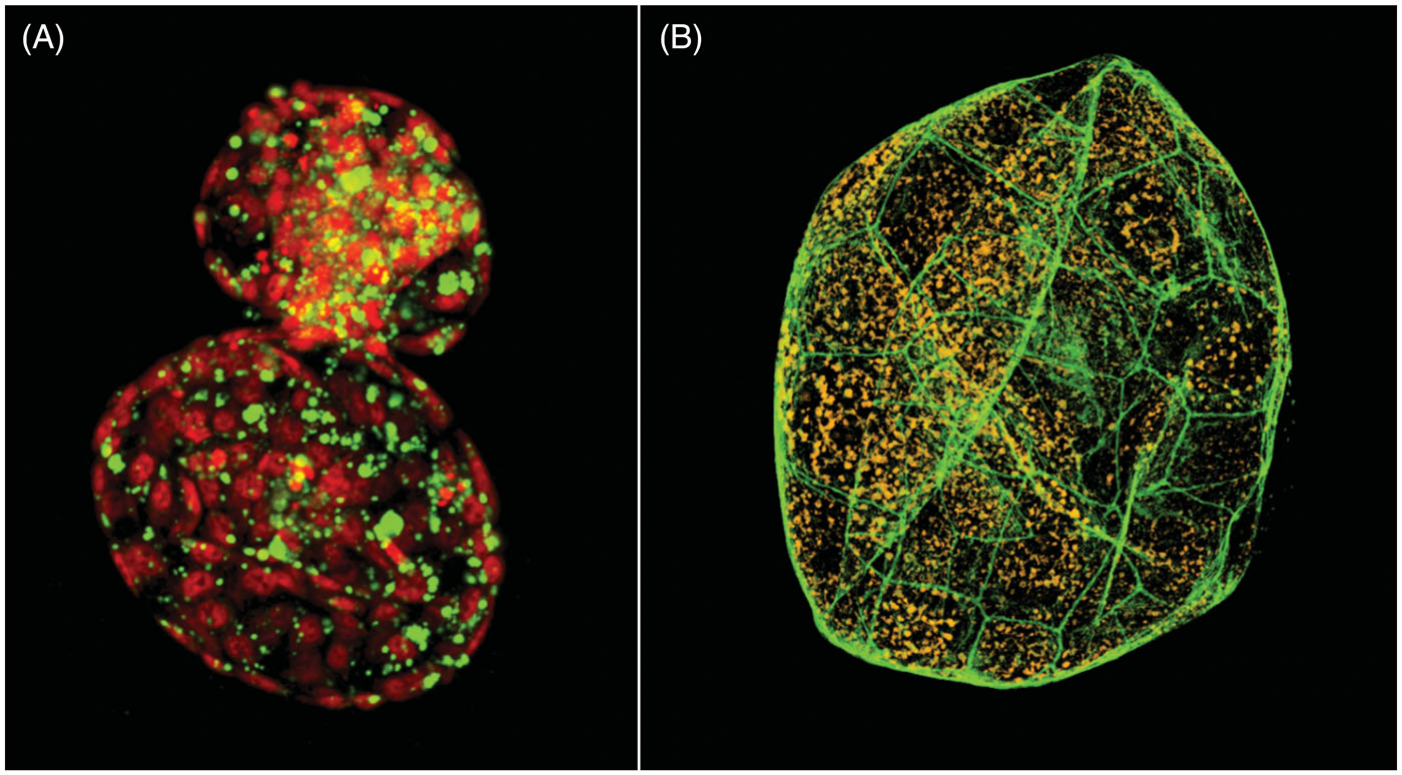
Three-dimensional images of embryos produced by confocal microscopy. (A) An *in vitro*-produced hatching bovine blastocyst 8 days after fertilisation. Visualised are nuclei (red) and neutral lipid droplets (green). (B) An *in vitro*-produced expanded porcine blastocyst 7 days after fertilisation. Visualised are f-actin (green) and active mitochondria (orange).

As regards bovines, genomic analyses have become a tool for selection of the most valuable individuals, already at embryo stages. This is one of the reasons for the massive increase in IVP within bovine production industry last years. The combination of embryo biopsies for genomic selection and cryopreservation of embryos has led to a very efficient procedure to improve genomic merit rapidly (70), a process that previously took several years. The genome-editing technologies mentioned above have also been adapted to cattle with good results. These technologies might help us understand and improve genetic traits in cattle as well as producing healthy cows that could provide us with bio-medical proteins in their milk (71). As with the pig, the cow oocyte maturation and early embryo development *in vitro* are valuable and sensitive models for humans, e.g. for investigations of the reproductive impact of environmental pollutants (3). Confocal microscopy is a method explored in the IVP facilities at SLU, Uppsala, and used for basic research and morphological characterisation of oocytes and embryos. Using fluorescent staining, we can easily assess various structures (Figure 3) and relate this phenotypic information to e.g. gene expression.

As cows are increasingly kept in warm parts of the world, we have to expect to see impaired reproductive performance in production animals not suited to hot environments. To perform IVP and transfer morula or blastocyst stages could be a future way to bypass heat stress, since the bovine embryo goes from being very sensitive to heat stress up to the four-cell stage, to largely resistant by the morula stage (72).

In many species, IVP media consisting of completely defined reagents or semi-defined reagents (usually including serum albumin) have been developed. This is attractive from a research perspective where all components can be accounted for and their functions assessed (55) but also from a biohazard point of view (33). Frozen *in vitro*-produced embryos are now transported all over the world. This is very positive from an animal welfare point of view, since it minimises the need for transportation of live animals to enhance the genetic progress, thereby increasing our possibilities to feed the growing human population. However, to ensure that this process does not contribute to the spreading of diseases, biosecurity must never be at risk.

The IVP systems must be enhanced and developed for more species for us to be able to preserve genetic diversity. When developing new methods, a better follow-up of the impact on the offspring is also needed to avoid situations like LOS. Animal welfare should always be assessed when introducing new technologies, including changes in medium compositions. The ultimate goal is the development of IVP systems that will produce embryos and offspring that are no different from those conceived and developed *in vivo*. We still have far to go, but comparative research and development across species in both animal and human embryology can provide us with the crucial knowledge needed. We can learn a lot from such comparative studies and thereby hopefully avoid unnecessary problems in the future.
